# An In Vitro and In Vivo Study of the Efficacy and Toxicity of Plant-Extract-Derived Silver Nanoparticles

**DOI:** 10.3390/jfb13020054

**Published:** 2022-05-10

**Authors:** Anjana S. Desai, Akanksha Singh, Zehra Edis, Samir Haj Bloukh, Prasanna Shah, Brajesh Pandey, Namita Agrawal, Neeru Bhagat

**Affiliations:** 1 Department of Applied Science, Symbiosis Institute of Technology, Symbiosis International (Deemed University), Pune 412115, India; desaianjana89@gmail.com (A.S.D.); bpandey@gmail.com (B.P.); 2 Department of Zoology, University of Delhi, New Delhi 110007, India; akankshasingh81293@gmail.com; 3 Department of Pharmaceutical Sciences, College of Pharmacy and Health Science, Ajman University, Ajman P.O. Box 346, United Arab Emirates; 4 Center of Medical and Bio-allied Health Sciences Research, Ajman University, Ajman P.O. Box 346, United Arab Emirates; s.bloukh@ajman.ac.ae; 5 Department of Clinical Sciences, College of Pharmacy and Health Science, Ajman University, Ajman P.O. Box 346, United Arab Emirates; 6 Department of Physics, Acropolis Institute of Technology and Research, Indore 453771, India; psnimc@gmail.com

**Keywords:** silver nanoparticles, characterizations, in vitro wound healing assay, in vivo *Drosophila* model, aloe vera, turmeric, biocompatibility, cytotoxicity

## Abstract

Silver nanoparticles (AgNPs) display unique plasmonic and antimicrobial properties, enabling them to be helpful in various industrial and consumer products. However, previous studies showed that the commercially acquired silver nanoparticles exhibit toxicity even in small doses. Hence, it was imperative to determine suitable synthesis techniques that are the most economical and least toxic to the environment and biological entities. Silver nanoparticles were synthesized using plant extracts and their physico-chemical properties were studied. A time-dependent in vitro study using HEK-293 cells and a dose-dependent in vivo study using a *Drosophila* model helped us to determine the correct synthesis routes. Through biological analyses, we found that silver nanoparticles’ cytotoxicity and wound-healing capacity depended on size, shape, and colloidal stability. Interestingly, we observed that out of all the synthesized AgNPs, the ones derived from the turmeric extract displayed excellent wound-healing capacity in the in vitro study. Furthermore, the same NPs exhibited the least toxic effects in an in vivo study of ingestion of these NPs enriched food in *Drosophila*, which showed no climbing disability in flies, even at a very high dose (250 mg/L) for 10 days. We propose that stabilizing agents played a superior role in establishing the bio-interaction of nanoparticles. Our study reported here verified that turmeric-extract-derived AgNPs displayed biocompatibility while exhibiting the least cytotoxicity.

## 1. Introduction

Engineered nanomaterials (ENMs) are widely incorporated into numerous technologies and consumer products owing to their remarkable plasmonic and optical properties. Physico-chemical properties, such as shape, size, surface charge, agglomeration, dispersity, and colloidal stability, influence the behavior of metal and metal oxides to a great extent. These nanoparticles are rapidly finding applications in diverse areas, including textile, cosmetics, agriculture, food packaging, pharmaceutical, chemical, engineering, and medical applications [1,2,3,4,5,6]. Metal and metal oxide nanoparticles, such as silver, gold, zinc oxide, and copper oxide, encompass diverse therapeutic applications, such as targeted drug release and antibacterial and antimicrobial activities, and are thus considered ideal candidates for wound dressings [7,8,9] and wound healing [10,11]. Metal nanoparticles prepared using plant extracts exhibit good antimicrobial activity, possibly due to the eco-friendly nature of the reactants as compared with chemically synthesized particles [12,13,14]. However, the intrinsic toxicity of metal NPs remains a disadvantage, which hinders their application in wound healing and hence demands further improvement in either the synthesis process or growth [15]. Among these metal NPs, silver nanoparticles (AgNPs) have a crucial role in wound healing due to their inherent antibacterial and anti-inflammatory properties [16]. Physical and chemical methods are mainly being used to synthesize metal nanoparticles, but the intrinsic toxicity of the nanoparticles synthesized by these methods is a matter of concern properties [17,18]. Cuts and burns cause damage to the epidermis, resulting in a wound. Open, unhealed wounds become sites for pathogen activity, causing severe infection and resulting in permanent damage or death [8]. Thus, it becomes imperative to heal the wound quickly to carry out the body’s normal functioning without microbial infection. The healing process helps minimize the scars and permits hard scabs to form [9]. Silver nanoparticles were discovered to be hazardous to cells, inhibiting cell growth and multiplication and triggering cell death depending on concentrations and exposure time [13,18]. It is evident that synthesis techniques play a major role in the preparation of less toxic nanoparticles. The biogenic mode of nanoparticle (NP) synthesis is gaining momentum due to the wide range of advantages it offers in economic viability, environmental sustainability, safety, and easy access to source materials [17]. The biogenic synthesis of AgNPs was achieved, and the antimicrobial activity of these NPs was demonstrated against many pathogens. In recent years, researchers used numerous plant extracts, such as *Moringa olifera*, coriander, ginger, turmeric, aloe vera, and neem [19,20]. Out of these, turmeric and aloe vera gained popularity because of their anti-inflammatory, anti-oxidative, and anti-neoplastic properties. Botanically, aloe vera is known as *Aloe barbadensis miller*; it is a perennial, xerophytic, succulent, shrubby or arborescent, pea-green hue plant that belongs to the *Asphodelaceae* (*Liliaceae*) family. There are 75 biologically active components, viz., vitamins (vitamin A, C, E, B12), enzymes (alkaline, alkaline phosphatase, amylase, bradykinase, carboxypeptidase, catalase, cellulose, lipase, and peroxidase), minerals (calcium, chromium, copper, selenium, magnesium, manganese, potassium, sodium and zinc), sugars (monosaccharides, polysaccharides), anthraquinones (laxatives), fatty acids (steroids, cholesterol, campesterol, β-sisosterol, and lupeol), hormones (auxins and gibberellins) and some amino acids [21,22]. The active ingredient present in turmeric is curcumin, botanically known as *Curcuma longa*, and is a member of the ginger family (*Zingiberaceae*). The mineral composition of turmeric is 0.63% phosphorous, 0.46% potassium, 0.20% calcium, and 0.05% iron. It was observed that elemental compositions of nitrogen (N), phosphorus (P), and potassium (K) are found to greater extents in saplings [23,24]. Researchers used various kinds of mammalian cell lines for in vitro study, but we chose human embryonic kidney (HEK-293) cells due to their easy growth and higher reproducibility. The scratch assay was conducted to study the toxicity in these cell lines. Amongst the different vertebrate models used for in vivo toxicity studies, *Drosophila* has gained wide attention because of its cost-effectiveness, shorter life cycle, ease of genetic manipulation, and ease of growth in laboratory conditions [25]. Developmental assays were done on the *Drosophila* model to study the behavioral pattern through toxic drug exposure. The aim of the present study was to increase the efficacy of wound-healing properties of nanosilver by using edible plants that are known in Ayurveda for their anti-bacterial, regenerative, and therapeutic properties. To achieve this, we used wholesome turmeric, and aloe vera leaves to synthesize silver nanoparticles because of the abovementioned properties of turmeric and aloe vera. The silver nanoparticles, synthesized with different reducing agents, were used to investigate the in vitro cytotoxicity in HEK-293 cell lines and the in vivo toxicity in a *Drosophila* model.

## 2. Materials and Methods

### 2.1. Preparation of the Biosynthesized AgNPs

Silver nitrate (AgNO_3_) solution from Sigma-Aldrich with a purity of 99.999% was used to prepare all silver nanoparticles. Four samples of AgNPs were synthesized using two different routes, viz., the chemical route (named AG-C), and using plant extracts, i.e., the biological route (AG-B). The AG-C sample was prepared by mixing 300 mL of 1 mM silver nitrate solution into 50 mL of 0.5 mM polyvinyl pyrrolidone (PVP) solution. The resulting solution was kept at 80 °C, and 0.5 M hydrazine hydrate solution was added drop by drop into it. This mixture was then kept at 80 °C for 1 h, resulting in the precursor for sample AG-C.

Two different plant extracts were used to synthesize the samples via biological routes. The relevant part of the plants was cleaned, crushed, and boiled in Milli-Q water and filtered to obtain the extracts described in detail elsewhere [26]. For the turmeric extract, dry turmeric roots bought from the market were used, and for the aloe vera extract, stems of the aloe vera plants grown in our pots were used. A 300 mL aqueous solution of 1 mM AgNO_3_ was mixed with different plant extracts, viz., aloe vera (A), turmeric (T), and a 1:1 mixture of aloe vera and turmeric (AT), to get a total of 500 mL of each AG-B precursor solution. The sample thus formed using aloe vera was named AG-BA and the sample formed using turmeric was named AG-BT. Similarly, the sample prepared using both aloe vera and turmeric extracts (1:1) was named AG-BAT.

The abovementioned precursors were initially kept at 80 °C for 1 h, then left at room temperature for a day to settle down in colloidal form and change color. The color change indicated the formation of nanoparticles. These colloids were then centrifuged at 5000 rpm for 20 min, filtered, and dried overnight in an oven at 50 °C to obtain powder samples.

### 2.2. Analytical Methods

The crystallographic structure of the prepared samples was studied using an X-ray diffraction (XRD) technique. A Bruker D8 Advance X-ray Diffractometer with Cu-Kα radiation (λ = 1.506 Å) was used to record X-ray diffraction (XRD) patterns. X-ray photoelectron spectroscopy (XPS) was done using XPS-SPECS GmbH, Germany, (Al K_α_ (1486.6 eV) X-rays). A field-emission scanning electron microscope (FESEM) was used to study the surface morphology. An FEI Nova NanoSEM 450 scanning electron microscope attached with Bruker X Flash 6130 with excellent energy resolution (123 eV for Mn Kα and 45 eV for Cu Kα) was used for the energy-dispersive X-ray spectroscopy (EDS). The transmission electron microscopy (TEM) images of silver nanoparticles were obtained with a TECHNAI20G2 transmission electron microscope.

All the AgNPs solutions were independently analyzed for their hydrodynamic diameter and polydispersity index on a Malvern Instrument Zetasizer Nano-ZS, Malvern (Malvern-Aimil Instruments private limited, New Delhi, India). The zeta potentials of the nanoparticles were also ascertained using the same instrument.

### 2.3. Biological Methods

The wound-healing property and cytotoxicity (in vitro) of the prepared AgNPs were studied using human embryonic kidney (HEK-293) cells. HEK-293 cells were cultured in a Petri dish and nourished using 10% fetal bovine serum along with Dulbecco modified eagle medium (DMEM). Inoculation was done with 200 µL of diluted (1:20) various AgNPs. The changes in the cells were captured using an optical microscope and the area covered by the cells was measured using the Image-J software. Details of the tests carried out are included in the Results section.

The toxicities of the AgNPs were tested on *Drosophila* fly stocks. All the assays were performed on wild-type *Drosophila* (Oregon-R), obtained from Bloomington *Drosophila* Stock Centre (BDSC), and conditions were maintained at 24.5 ± 0.5 °C, 60% humidity, and a 12 h light cycle.

A stock of 5% (*w*/*v*) AgNPs (all types) suspension was sonicated (QSONICA Sonicators) for 30 min at an amplitude of 30 to obtain a homogenous suspension. The final concentrations of 25 mg/L, 50 mg/L, and 250 mg/L were obtained by vigorously mixing different AgNPs in partially cooled fly food. The nanoparticles and the dose used were AG-C (25 mg/L, 50 mg/L), AG-BT (25 mg/L, 50 mg/L, 250 mg/L), AG-BA (25 mg/L, 50 mg/L), and AG-BAT (25 mg/L, 50 mg/L). Further details of various tests and studies are included in the Results section.

### 2.4. Statistical Analysis

All the plotted values of in vivo represent mean + SEM (standard error of the mean), and error bars represent the positive SEM value. Statistical analysis was carried out for all the assays using a two-tailed independent Student’s *t*-test for pairwise comparisons. The significance level was ascribed at 0.05. Regarding *p*-values, *** *p* < 0.001, ** *p* = 0.001–0.01, and * *p* = 0.01–0.05.

## 3. Results

### 3.1. Physicochemical Characterization

#### 3.1.1. XRD and EDS

XRD and EDS were utilized to study the morphology and composition of the samples.

**Figure 1:**
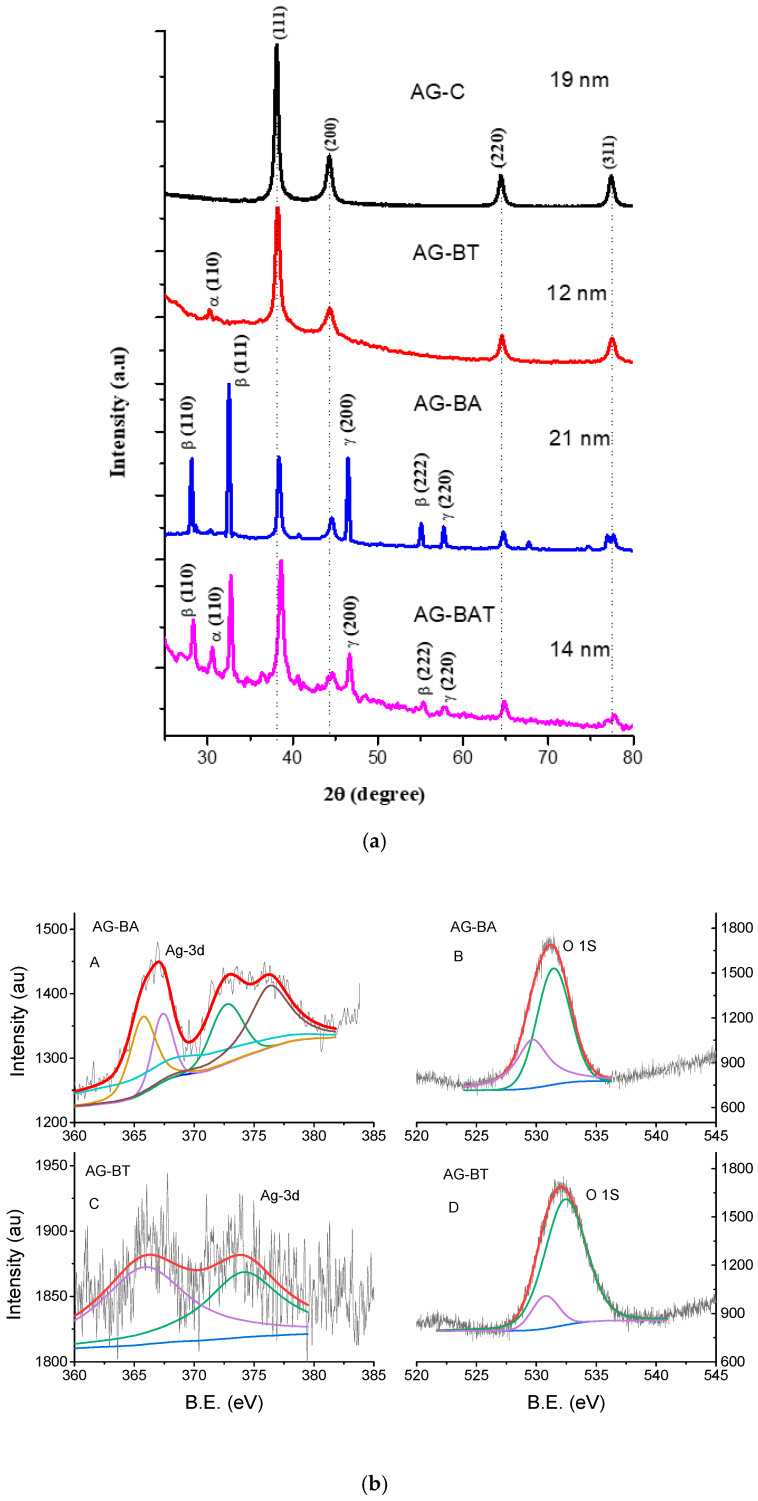
(**a**) X-ray diffraction patterns of different AgNPs samples. Dotted lines are drawn to show peaks corresponding to pure Ag. Various peaks in the pattern correspond to different phases of silver oxide, viz., α-Ag_4_O_4_, β-Ag_2_O, and γ-Ag_3_O_4_. (**b**) XPS binding energy spectra of samples AG-BA and AG-BT: (**A**,**C**) silver Ag 3d spectra and (**B**,**D**) oxygen with a high resolution.

Figure 1 shows the X-ray diffraction patterns of all the synthesized silver nanoparticles. The X-ray diffraction (XRD) pattern of silver nanoparticles prepared using PVP (AG-C) showed a pure phase of silver.

XRD patterns of nanoparticles synthesized with turmeric extract (AG-BT) and both aloe vera and turmeric extracts (AG-BAT) displayed oxide peaks corresponding to Ag_4_O_4_ (α) (silver I, III) (also known as Ag_2_O_2_) [27].

The silver nanoparticles prepared using aloe vera extract (AG-BA) and using both aloe vera and turmeric extracts (AG-BAT) showed the presence of silver oxide phases, such as Ag_3_O_4_(γ) and Ag_2_O(β) [28,29]. All these peaks were matched with JCPDS data and indexed. A few other peaks were also noticed in this diffractogram. These might have been due to the in situ formation of some unstable oxides of silver. The average particle/crystallite size as calculated by Scherer’s formula was 19 nm for AG-C, 12 nm for AG-BT, 21 nm for AG-BA, and 14 nm for AG-BAT. The Scherrer formula is the standard formula used for calculating the crystallite size and is given by

(1) $$\tau =\frac{K\lambda }{\beta \;cos\theta }$$

where *τ* is the crystallite size, *K* is the dimensionless shape factor with a value of 0.9, *λ* is the wavelength of the incident X-rays (1.506 Å), *β* is the full width at half maximum of the strongest peak, and *θ* is the Bragg’s angle at which the peak is situated.

One can also observe there was a slight shift in the peak positions toward higher angles in AG-BAT. This indicated stress in the sample, which could have been introduced due to the formation of different silver oxides of different sizes.

Some oxides of silver are quite elusive in XRD. Nevertheless, their presence was indicative in other tests. Hence, to correctly identify and claim their presence in our samples, we performed XPS. XPS gives the oxidation states of elements by giving their binding energy values (BE). Pure silver shows narrow peaks corresponding to Ag(3d_5/2_) at 368.5 eV and Ag(3d_3/2_) at 374.5 eV, and the oxygen O 1s peak is observed at 530.5 eV. It is known that when silver forms an oxide, it lowers the Ag(3d) binding energies [30] and increases the O 1s binding energy. Oxides also lead to the widening of the peaks (i.e., FWHM) [31]. Figure 1b represents the fitted XPS data of our samples AG-BA and AG-BT. In the sample AG-BA, we observed the fitted peaks O 1s corresponding to 529.8 eV and 531.46 eV and the Ag peaks at 376.3 eV, 372.7 eV, 367.4 eV, 366.8 eV, and 365.7 eV. As per the literature, these peaks correspond to metallic silver (376.3 eV, 372.7 eV), Ag (I)—367.4 eV, Ag (III)—366.8 eV, and Ag (II)—365.7 eV oxidation states. The O 1s BE values corresponding to 529.8 eV indicated the presence of Ag/Ag_2_O and that between 530–532 eV showed the presence of superoxides, carbonates, or hydroxyl ions [32,33,34]. No peaks of carbonates were detected in the XRD; hence, their presence in our samples was ruled out by us. The sample AG-BA showed more toxicity; hence, it is safe to say that this sample contained more superoxide than hydroxyl ions. The presence of these binding energy peaks was indicative of the presence of AgNPs, Ag_2_O, and Ag_3_O_4_ in the prepared sample AG-BA. In the sample AG-BT, we observed O 1s peaks corresponding to BE 530.2 eV (AgO or silver (I, III)) and 532.38 eV. The peak at 532.38 eV may have been because of the presence of superoxide, leading to increased toxicity or, as per some studies, may reflect the presence of contamination [35], which in our case could have been an outcome of biological synthesis. The Ag 3d binding energy values observed in the sample were 374.08 eV and 366.1 eV corresponding to Ag and Ag (III) [36]. These observations confirmed the presence of mainly AgNPs and a small amount of Ag_4_O_4_ (silver (I, III)) in this sample. These silver oxides came up in our samples due to the process of synthesis, which involved interaction with a biological environment. This interaction led to the oxidation of silver, which would normally be absent in chemically capped nanoparticles [24,37].

**Figure 2:**
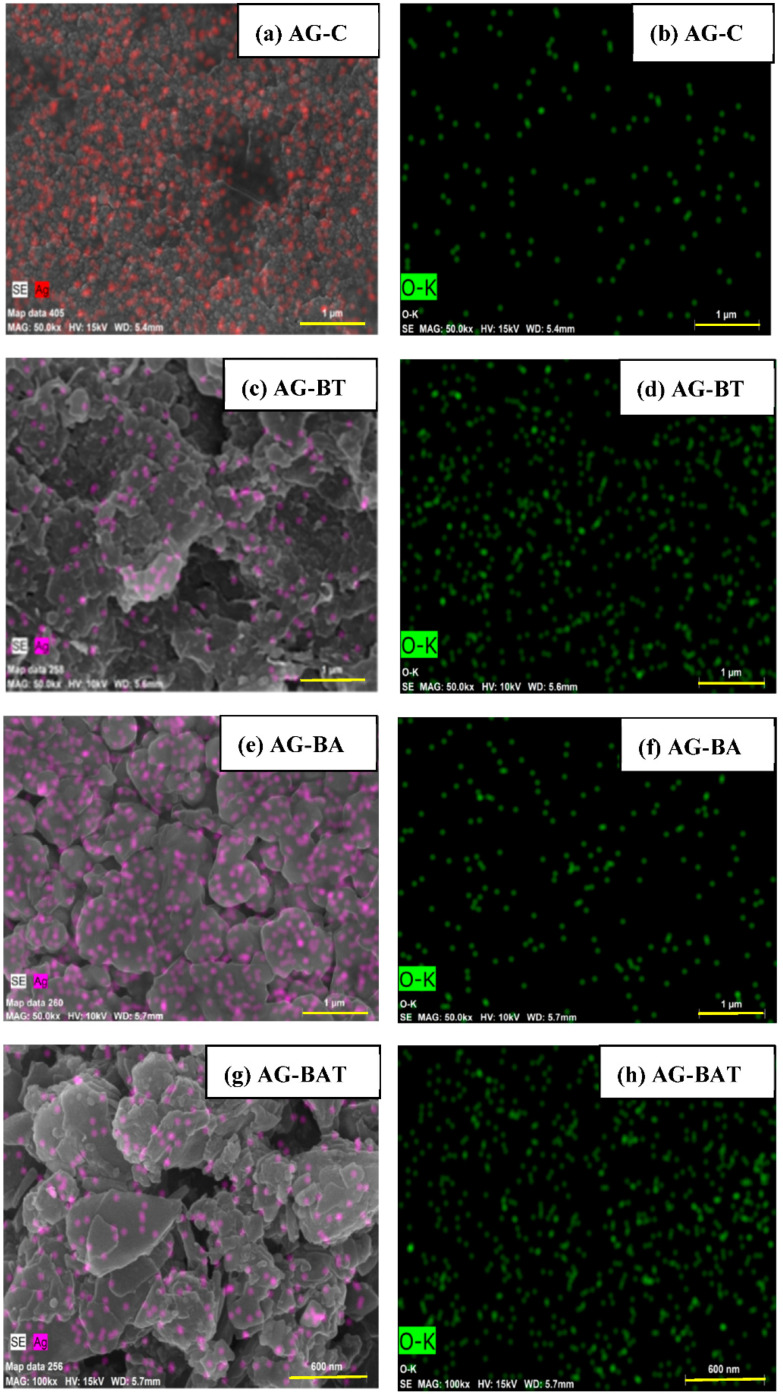
EDS images of AgNPs showing: elemental silver in the (**a**) chemically synthesized silver, (**c**) synthesis achieved using turmeric extract, (**e**) synthesis achieved using aloe vera extract, and (**g**) synthesis achieved using aloe vera and turmeric extracts in equal proportion; elemental oxygen (**b**,**d**,**f**,**h**) in each sample, respectively.

The energy-dispersive X-ray spectroscopic (EDS) technique was used to determine the composition of various elements in the synthesized AgNPs. All samples showed some amount of elemental oxygen. The XRD pattern of AG-C was not able to reflect the formation of oxides. There were oxide peaks observed in the XRD pattern of samples prepared in the presence of the aloe vera extract, but a very small fraction of oxide peaks was observed in the silver prepared using the turmeric extract. It is interesting to note that the elemental analysis gave almost equal amounts of oxygen in the samples prepared in the presence of turmeric. This indicated that the presence of turmeric extract restricted the formation of oxides and trapped a lot of free oxygen in the silver nanoparticles. The EDS studies, combined with the XRD, confirmed silver oxides in the samples prepared using the aloe vera extract. The presence of elemental silver and elemental oxide in each sample can be seen in the EDS images (Figure 2) and the amounts are presented in Table 1.

It was evident from the EDS results that the elemental silver in AG-BA and AG-C was much higher than that in AG-BT and AG-BAT (Figure 2).

#### 3.1.2. Field-Emission Scanning Electron Microscope (FESEM) Results

**Figure 3:**
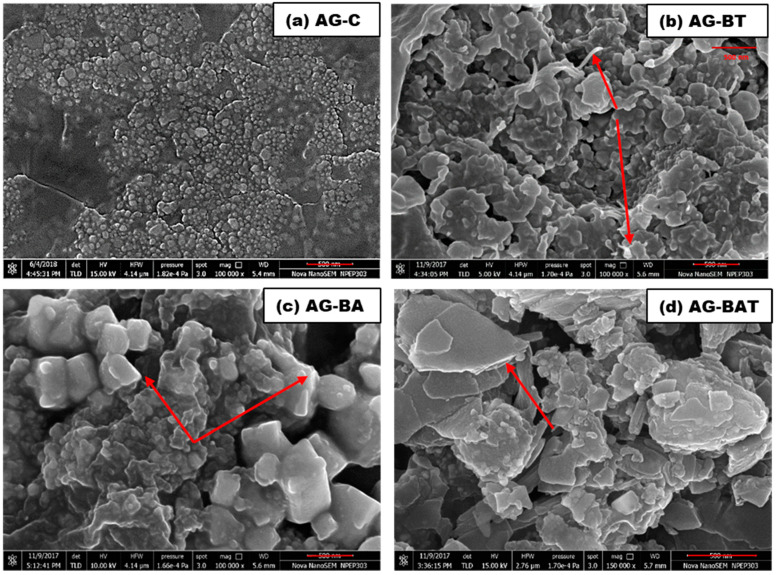
FESEM micrographs of differentially synthesized AgNPs: (**a**) AG-C, (**b**) AG-BT, (**c**) AG-BA, and (**d**) AG-BAT. The arrows indicate some oxygen-rich regions. The bar shown under each image depicts 500 nm.

The surfaces of the chemically synthesized silver nanoparticles (AG-C) showed monodispersed spherical granular particles, with some patches of agglomeration (Figure 3).

A predominant arrowhead or pointed rod-like structures mixed with flakes and clusters were formed when turmeric extract (AG-BT) was used (Figure 3b).

On the other hand, the AgNPs prepared using aloe vera extract (AG-BA) showed cubic pillar-like monoclinic structures (Figure 3c). It is also evident from the FESEM images that this structure was monoclinic (related to Ag_3_O_4_) and some areas had a low melting point. Almost all the corners and edges were melted due to the energy of the electron beam. When both extracts were used (AG-BAT), the surface morphology was completely different as compared with the other samples and had layered flakes (2D structures) (Figure 3d). Shiny regions observed in the FESEM micrographs indicated the presence of oxide-rich regions. XRD analysis gave an indication of the type of oxides formed in the three samples synthesized using plant extract. There were some common silver oxides that can be seen in AG-BA and AG-BAT. In the sample AG-BAT, the shining edges might have been because of the presence of Ag_3_O_4_. Higher magnification was achieved due to higher electron energy. Silver oxides other than Ag_3_O_4_ had lower melting points, and hence, tended to melt at lower temperatures obtained using high electron energy. Since Ag_3_O_4_ has a relatively high melting point (1605 °C), it does not melt easily and gave shiny edges in the FESEM images. It is interesting to note that the silver nanoparticles synthesized in the presence of turmeric extracts led to flake-like structures. The release of oxygen is easier in flake-like structures and may be useful in applications where free oxygen is helpful in healing.

#### 3.1.3. TEM and SAED

**Figure 4:**
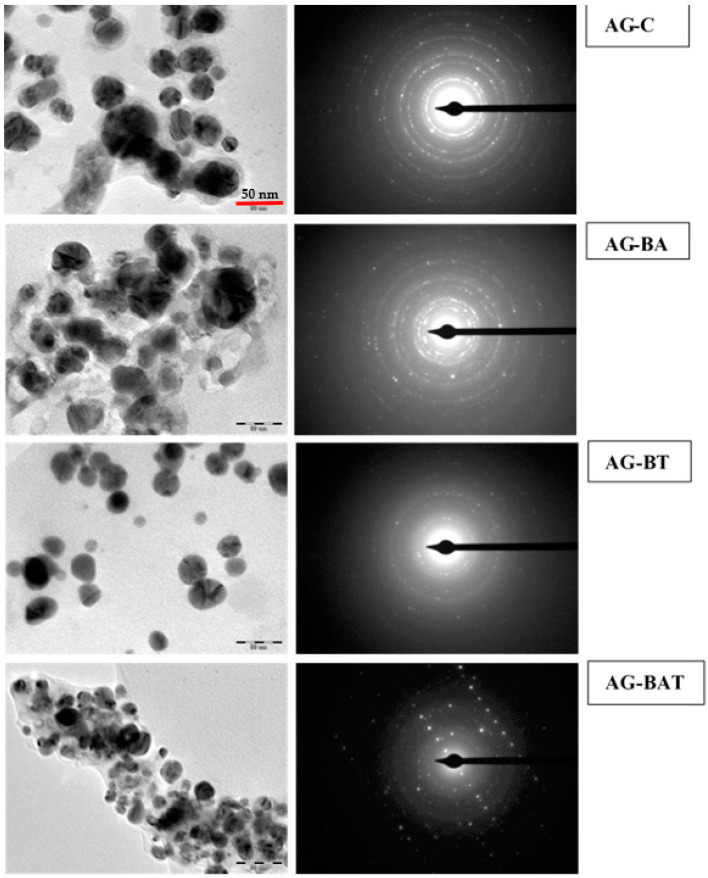
TEM micrographs and SAED patterns of silver nanoparticles. All TEM micrographs are of equal magnification.

Transmission electron micrographs with the corresponding SAED patterns of all the synthesized silver nanoparticles are shown in Figure 4.

The Ag-C sample showed mainly capped and crystalline spherical nanoparticles [38].

The structure of the silver nanoparticles was quite distinct when synthesized using turmeric extracts. Core–shell-type spherical nanoparticles with an almost uniform distribution were observed by Chircov et al. in their study of iron oxide and were reported with the help of SAED patterns as well [39]. It was also observed that when these NPs are used for healing purposes (scratch test), this led to better healing properties than others. One reason could be due to the oxygen trapped in the core–shells, and when they interact with live tissue, they release O_2_, which helps with the growth of cells.

The shapes of the nanoparticles were irregular and fussed when synthesis was done in presence of aloe vera. When both turmeric and aloe vera were used we obtained non-uniform (also evident by PDI > 0.7 in Table 2) hollow spherical particles [40]. It is interesting to note that only by changing the medium of synthesis; nanoparticles with different shapes were produced.

#### 3.1.4. Dynamic Light Scattering (DLS) and Zeta Potential Measurements

Dynamic light scattering (DLS) measures the Brownian motion of particles in a mixture and gives the size distribution based on intensity fluctuations.

It is important to note that DLS measures the size distribution of clustered particles and, therefore, shows the tendency of particles to agglomerate in the colloidal form. The average hydrodynamic diameters of AG-C, AG-BT, AG-BAT, and AG-BA as determined by DLS were 124.4 nm, 494.5 nm, 778.9 nm, and 463.2 nm, respectively (Table 2).

Apart from this, all the nanoparticles assessed displayed a polydispersity index below 1 and, therefore, highlighted their monodispersity and lower tendency to agglomerate in the solution (Table 2).

AG-BT displayed good colloidal stability and exhibited a zeta potential of −28.9 mV. AG-BAT and AG-BA displayed moderate colloidal stability, exhibiting zeta potentials of −23.7 mV and −15.35 mV, respectively [41].

Contrarily, AG-C was highly unstable in the colloidal solution, displaying a zeta potential of −4.84 mV. It was evident from our study that the use of turmeric extract for the synthesis of NPs led to greater colloidal stability and uniformity in the size of nanoparticles (monodispersity).

### 3.2. Biological Studies

#### 3.2.1. In Vitro Studies

The human embryonic kidney (HEK-293) cells were seeded in nine-well culture plates (seed density (3–6) × 10^5^ cells/cm^3^). In order to produce a scratch in a confluent monolayer, the 200 µL sterile pipette tip was used. Scratches were made one at a time with constant pressure so that the same scratch width was maintained throughout the plates. The cells were washed extensively with phosphate-buffered saline (PBS) to remove the detached cells and debris. The prepared samples, along with the A and T extracts, were inoculated in nine-well culture plates. The images were observed and captured with the help of an inverted optical microscope with an attached camera. All the Petri dishes were observed for 2 days at the same time after inoculation. These studies were done thrice and the average of three sets of the assay was considered for evaluation.

The ‘wound healing test’ or ‘scratch test’ is the most common laboratory test performed to study the 2D cell migration, cell-to-cell interaction, cell proliferation, etc. Many commercially available creams and ointments used for healing surface wounds contain silver. Hence it is important to study the effect of silver nanoparticles in cell migration (tissue migration), as the wounds heal mainly via this process. Some nanoparticles are also known to cause cell death via apoptosis [42].

**Figure 5:**
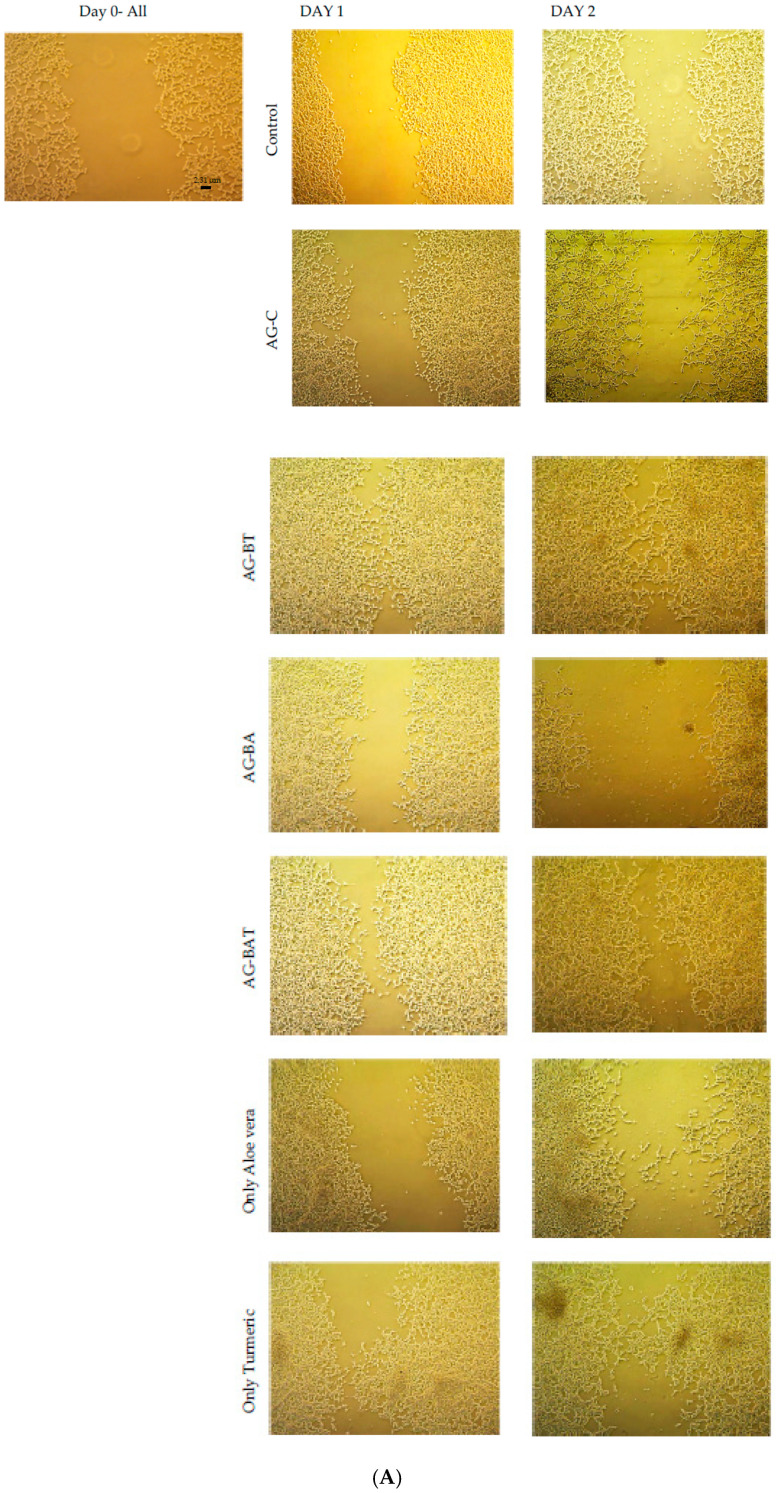

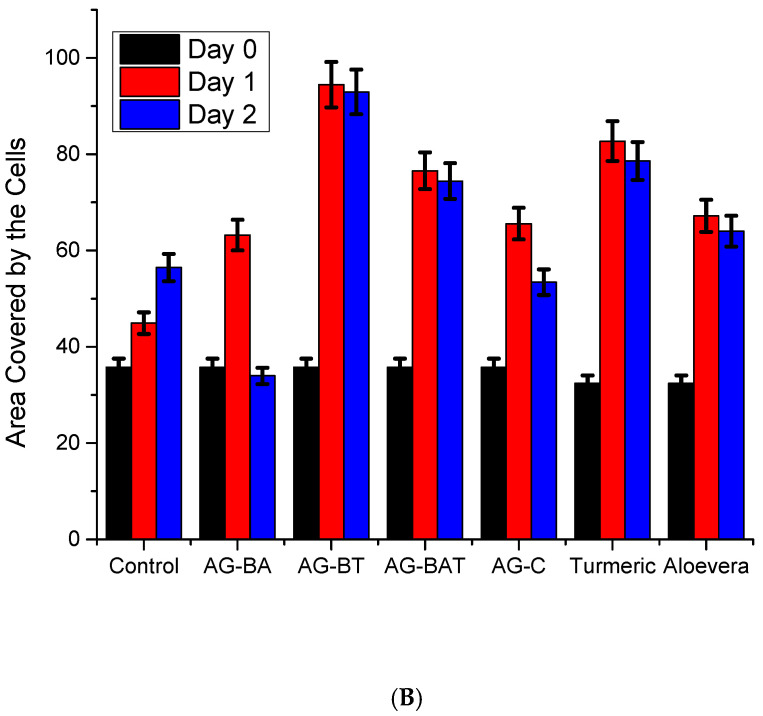
(**A**) Images from the scratch test analysis; (**B**) quantitative representation of cell growth measured on day 1 and day 2 using all the synthesized samples, as well as the plant extracts, in comparison with the control. Error: ±5%.

Hence, studying an in vitro ‘scratch test’ is imperative and was achieved by inoculating the medium with the various synthesized nanoparticles. The wound-healing capability of AgNPs was assessed upon the inoculation of the scratched cell culture with different samples for AgNPs for 2 days. On day 1 post-inoculation, wound healing (marked by the growth of cells near the scratch) was higher in all the dishes containing AgNPs than in the control dish (Figure 5A (actual pictures of the cells), Figure 5B (graphical depiction of the area covered by cell growth)).

On day 2, however, cells inoculated with AG-C and AG-BA showed extensive death (Figure 5A). Cell cultures inoculated with AG-BT and AG-BAT exhibited almost full wound closure, with significant cell proliferation (Figure 5A). From the figures, the effect of just the plant extracts on cell growth can also be observed. It is evident that though the turmeric extract alone also enhanced the cell growth, it was to a lesser extent as compared with AG-BT. This shows that the presence of the AgNPs played an important role in wound healing capacity and is explained in detail later in the Discussion section. On the other hand, the aloe vera extract alone showed higher healing potential as compared with AG-BA.

To ascertain the role of aloe vera and turmeric extracts alone on cell proliferation, we also undertook the scratch test using just the extracts. Our results showed that the aloe vera extract, in its current concentration, was on par with AG-C on day 1 and better than the control and AG-BA in terms of its wound healing properties. However, the extract showed ineffectiveness in further cell growth on day 2 but did not show toxicity either. Similar observations were made for the turmeric extract, yet better than the aloe vera extract.

Though the aloe vera extract, turmeric extract, AG-BAT, and AG-BT all showed reduced or negligible cytotoxicity, amongst all samples tested, AG-BT fared best and enhanced the wound-healing capacity of cells with accelerated growth, while AG-C and AG-BA proved to be cytotoxic.

#### 3.2.2. In Vivo Studies

##### Analysis of Larval Development and Adult Eclosion

The 4–5 days old parental wild-type flies were allowed to lay eggs in a chamber overnight, and 75 eggs were transferred to each vial containing media supplemented with and without different doses of various nanoparticles. Pupation success was evaluated by counting the number of viable pupae formed out of the 75 eggs transferred to a vial.

Similarly, the adult eclosion percentage from each condition was estimated by scoring the total number of F1 flies that eclosed from the vials of that condition.

Five vials per condition were scored for pupation and eclosion assays [25,43].

##### Climbing Assay

A climbing assay was performed on the eclosed F1 flies from and aged upon AgNP-treated fly food on days 10 and 30. Climbing ability was assessed by monitoring the vertical climbing of flies in an empty glass tube (diameter 2.2 cm) with marked gradations.

A group of 10 flies in two replicates per condition were transferred to a glass tube and acclimatized, and then we tapped to the bottom of the tube and allowed them to climb.

The number of flies passing the 10 cm mark in 15 s was recorded for each condition. Three trials per replicate condition were allowed and noted [25].

##### Pupation and Eclosion Rates of Flies Exposed to AgNPs

To ascertain the effect of ingestion of various nanoparticles on developmental viability at pupal and adult stages, we comprehensively analyzed the pupation rate and eclosion success in *Drosophila*. The larvae were fed with AG-C (25 mg/L, *p* < 0.001; 50 mg/L, *p* < 0.001), AG-BA (25 mg/L, *p* = 0.008), AG-BT (50 mg/L, *p* < 0.001; 250 mg/L, *p* = 0.002), and AG-BAT (25 mg/L, *p* < 0.001; 50 mg/L, *p* = 0.007).

**Figure 6:**
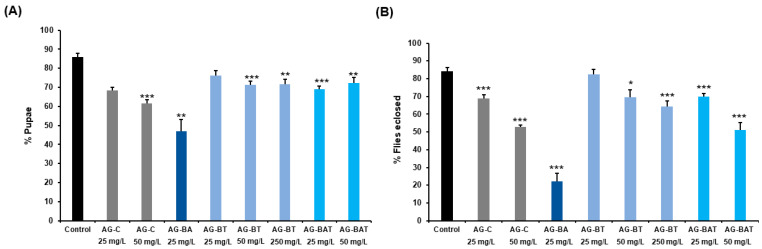
(**A**) Percentage of pupae formation; (**B**) adult eclosion assessed in each condition. Values represent mean ± SEM. *p*-value: *** *p* < 0.001; ** *p* = 0.001–0.01; * *p* = 0.01–0.05. Statistical analysis was done by Student’s *t*-test.

The percentage of larvae successfully pupating upon ingestion of the AgNPs was substantially lower than the larvae reared on standard food. However, it is worth noting that the pupation percentage in larvae reared with AG-BT (25 mg/L, *p* = 0.013) was comparable to the control (Figure 6A).

Significant lethality witnessed in most AgNP-treated conditions highlighted the extent of toxicity caused by nanoparticle ingestion from early developmental stages. The toxicity observed was the lowest in larvae that ingested 25 mg/L AG-BT, indicating that the AgNPs synthesized using turmeric extract were potentially safer than nanoparticles derived chemically or with other plant extracts.

Larvae reared on 50 mg/L AG-BA (aloe-derived AgNPs) died very early in development, did not pupate, and hence, are not presented in the graph.

Eclosion observed for the same doses of AgNPs showed significantly lower flies eclosing from AG-C (25 mg/L), AG-BA (25 mg/L), AG-BT (50 mg/L, 250 mg/L), and AG-BAT (25 mg/L, 50 mg/L) as compared with the control condition. Flies eclosing from AG-BT (25 mg/L) displayed the same eclosion percentage as the control (*p* = 0.595) (Figure 6B).

Conclusively, our results indicated that 25 mg/L of turmeric-derived AgNPs (AG-BT) was found to be compatible with developmental survival.

##### Cuticular Melanization of Flies Exposed to AgNPs

Cuticular pigmentation is a critical parameter of an insect’s life, as it influences various physiological and behavioral aspects, including lifespan, immunity, stress tolerance, and courtship. Loss of cuticular melanization is a well-known manifestation of AgNPs exposure in *Drosophila* [44,45,46]. Importantly, it was observed that the extent of loss of pigmentation in flies upon NP ingestion is directly correlated with decreased longevity, decreased locomotor activity, and overall toxicity.

To assess phenotypic aberrations in flies ingesting differentially synthesized silver nanoparticles, we monitored cuticular melanization in the pupal and adult stages.

**Figure 7:**
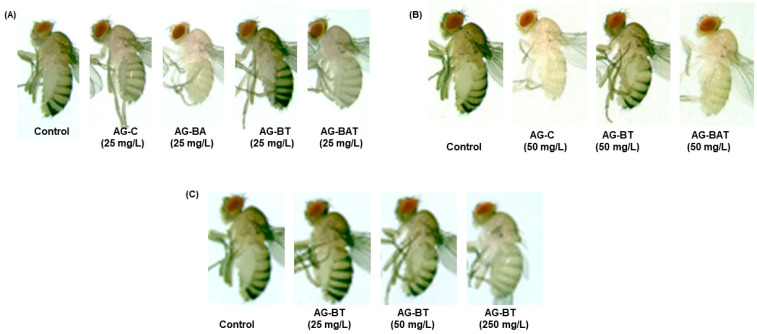
Cuticular melanization of the pupae formed upon exposure to (**A**) nanoparticles at 25 mg/L, (**B**) nanoparticles at 50 mg/L, and (**C**) turmeric-derived nanoparticles at different concentrations.

**Figure 8:**
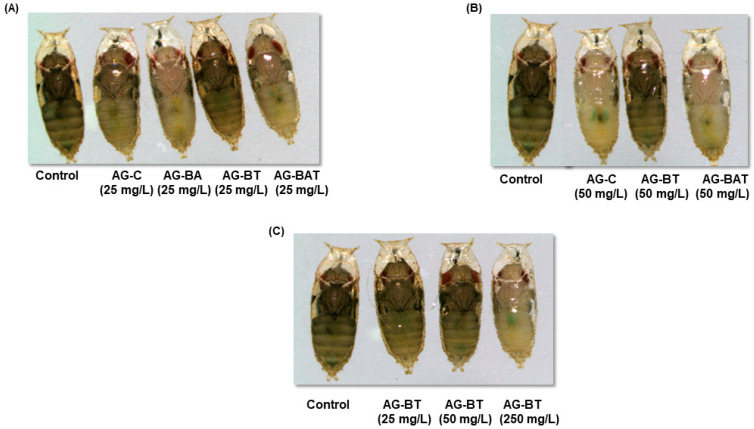
Dose-related effect of nanoparticle ingestion on cuticular melanization of adults: (**A**) 25 mg/L fed nanoparticles, (**B**) 50 mg/L ingested nanoparticles, and (**C**) ingested AG-BT nanoparticles at different concentrations.

We observed a similar degree of demelanization in pupae and adults reared upon equal doses of AG-C and AG-BAT (i.e., 25 mg/L AG-C = 25 mg/L AG-BAT and 50 mg/L AG-C = 50 mg/L AG-BAT) (Figure 7 and Figure 8).

While pupae and flies reared upon 25 mg/L AG-BA displayed the maximum loss of pigmentation as compared with those reared on other nanoparticles at the same dose, larvae reared upon 50 mg/L AG-BA did not pupate and hence could not be assessed for any further assays. Remarkably, pupae and adult flies reared on an AG-BT-enriched diet did not lose cuticular melanization at 25 mg/L and 50 mg/L and displayed loss of melanization only at a very high dose of 250 mg/L (Figure 7C and Figure 8C).

It is generally accepted that the extent of AgNP-mediated loss of melanization is proportional to the extent of system dysfunctions observed in *Drosophila* [44,45].

These results suggest that AG-BT exerted the least toxicity on cuticular pigmentation of all the assessed nanoparticles and did not interfere with the melanin synthesis pathway up to the concentration of 50 mg/L.

##### Impact of AgNPs on Degree of Climbing of Flies

Further, to explore the impact of ingestion of various AgNPs supplemented food on *Drosophila*, we monitored the crucial vertical climbing behavior and survival of adults for 30 days. The climbing assay is widely used in neurodegenerative fly models and the degree of climbing defect directly correlates to the degree of neuronal atrophy (or toxicity).

**Figure 9:**
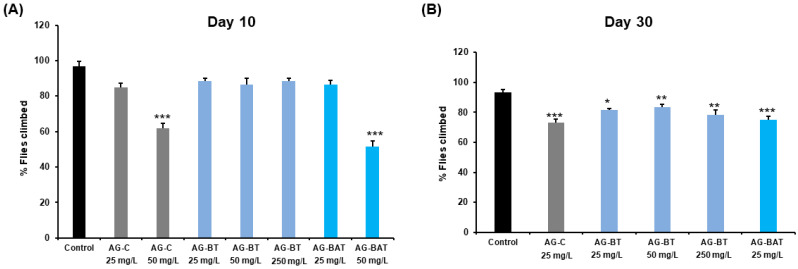
Assessment of the climbing ability of flies upon exposure to different nanoparticles. The climbing ability (**A**) of 10-day-old flies was compromised at 50 mg/L concentrated ingestion of AG-C and AG-BAT nanoparticles, and (**B**) was affected in all the surviving conditions at day 30 to various degrees. Values represent mean + SEM. *p*-value: *** *p* < 0.001; ** *p* = 0.001–0.01; * *p* = 0.01–0.05. Statistical analysis was done using Student’s *t*-test.

As the ingestion of AgNPs was reported to cause severe impedance in climbing ability at different concentrations, we evaluated the extent of climbing disability in various experimental conditions with aging. On day 10, the climbing ability was significantly reduced in flies reared upon 50 mg/L of AG-C and AG-BAT while remaining comparable to the control in all other feeding conditions (Figure 9A).

By day 30, the vertical climbing ability declined in all the adults exposed to different doses and types of AgNPs with maximum impairment seen upon aging on AG-C and AG-BAT at 25 mg/L and least impedance observed in AG-BT 25 mg/L raised adults (Figure 9B).

Notably, flies fed with and aged upon 50 mg/L AG-C and AG-BAT did not survive up to day 30 and hence could not be monitored for climbing ability.

Conclusively, while on day 10, no climbing disability was observed in flies reared upon AG-BT-enriched food, even at very high dose ingestion (250 mg/L), the climbing ability was reduced by day 30 in a dose-dependent manner in all the treated conditions.

## 4. Discussion

Despite a plethora of studies depicting the characteristics, assimilation, behavior, and metabolism of nanoparticles in living systems, ambiguity regarding their cytotoxicity persists and presents challenges regarding their application. It was been earlier reported by Olga et al. [47] that the smaller the size of the AgNPs, the greater the cytotoxicity; moreover, Ag nanoparticles, being neutral, show less cytotoxicity as compared with Ag ions. Further, Hamouda et al. reported that some biosynthesized AgNPs are less cytotoxic on certain human cell lines, such as MCF-7 and HCT-116 [48].

To further explore the toxic potency, we comprehensively analyzed the physicochemical characteristics of chemically synthesized and biosynthesized AgNPs and tested them in vitro and in vivo.

Much of the cytotoxicity encompassing AgNPs was shown to result from its conversion to Ag+ ions intracellularly.

It was shown earlier that the biogenic nanoparticles exhibit less toxicity in vitro. Cytotoxicity of nanoparticles is mainly attributed to the generation of reactive oxygen species (ROS), which further goes on to activate inflammatory and apoptotic pathways [49]. ROS plays an important physiological role in cell proliferation, repair, and signaling at low levels. However, when the levels of ROS increase beyond a threshold, the cells undergo oxidative stress, which eventually leads to cell death.

The interaction of ROS (H_2_O_2_) with silver nanoparticles, explained by Asharani et al. [50], is as follows:

2Ag + H_2_O_2_ + 2H^+^ → 2Ag^+^ + 2H_2_O (2)

and according to Di He et al. [51]:

Ag (NP) + H_2_O_2_ → Ag^+^ + O^2●−^ + H_2_O (3)

From the scratch test, similar behavior was observed for AG-C and AG-BA. The common observation was that both these samples initially (day 1) seemed to help in cell growth and then later (day 2), caused the death of the cells.

In comparison, the amount of cell death was greater with AG-BA. The initial growth and subsequent death were observed because cell viability depends on the concentration of nanoparticles and time. The initial growth of the cells indicated that the ROS produced by the interaction of the cell organelle with the Ag+ ions were low in concentration. Low levels of ROS are known to aid the growth of cells [52]. However, as time progresses, the amount of ROS produced increases leading to cell death by apoptosis. In accordance with the earlier studies [16,49], higher toxicity in AG-BA is due to its larger size, a higher concentration of elemental silver, and the existence of significant amounts of hazardous oxide (Ag_2_O). Similarly, these oxides may also interact with the SOD (H_2_O_2_) (SOD—superoxide dismutase) inside the cell to produce Ag+ ions.

From the above discussion, it is clear that Ag+ ions are the prime agents for producing highly toxic ROS, both extracellular and intracellular in the presence of Ag_2_O.

In contrast, the non-toxicity and enhanced cell viability shown in samples inoculated with AG-BT and lower toxicity observed for AG-BAT could be attributed to the lower concentration of elemental silver [16,53,54,55,56], smaller size [52,53,57], spherical shape, and higher colloidal stability (zeta potential report) of these AgNPs [16,52,53]. A lower amount of AgNPs leads to low levels of H_2_O_2_ (ROS) and smaller AgNPs increase extracellular superoxide dismutase (SOD_3_). This helps in cell growth and cell proliferation and decreases apoptosis. Earlier studies also suggested that increased oxygen levels in cell culture led to lower oxidative stress and lower ROS production [58]. It was also evident from the pointed rod-like structure seen in the FESEM (Figure 3) that this was rich in oxygen (the bright tip), which might easily release oxygen and increase the concentration of dissolved oxygen in the cell culture. This oxygen-releasing theory was further verified by the core–shell/hollow structures of AG-BT and AG-BAT, as seen in the TEM images.

The core–shell/hollow structure might release trapped oxygen while interacting with the cellular environment. Though most silver oxides were reported to dissolve in the cell environment, Ag_4_O_4_ (the oxide present in AG-BT and AG-BAT) is stable, and hence does not dissolve to give Ag+. The absence of Ag+ ions in AG-BT reduces the risk of cytotoxicity.

The effect of Ag_4_O_4_ is important, as its small presence in the sample AG-BAT seemed to influence its behavior and overcome the effect of Ag_2_O, thus making it less toxic compared with AG-BA. AG-C may also owe its increased toxicity to its instability, which is clear from our DLS studies. In contrast, the zeta potential data confirmed the high colloidal stability for AG-BT in water.

Therefore, our in vitro results highlighted the wound-healing and cell proliferative effects of turmeric-derived AgNPs, which may have largely been due to the ability of turmeric to quench excess ROS and its compatible physicochemical properties with the biological system.

We found a substantial impact of AgNPs ingestion on larval development, as was evidenced by a significant decrease in the formation of viable pupae in the treated conditions. Out of all the nanoparticles assessed, aloe-derived AgNPs ingestion caused the most detrimental impact on larval development and pupal formation, as the larvae died at a moderate dose (50 mg/L) exposure and did not pupate, while those reared at 25 mg/L displayed a significant reduction in pupation and died within 2 days of eclosion. This inherent toxicity associated with aloe-derived AgNPs may have been due to the presence of two silver oxide phases in the NPS, as ascertained using XRD, XPS, and EDS. The eclosion percentage of flies was also significantly reduced upon exposure to different AgNPs. The AgNPs are known to cause endocrine disruption in various model organisms [59] and may act through disruption of ecdysone and juvenile hormone (JH) to impede developmental progress. Induction of oxidative stress and apoptosis was also evidenced upon exposure to AgNPs in larval and adult tissues [46,60]. At the same time, phytoconstituents of turmeric, especially curcumin, possess well-known anti-oxidative activity. Therefore, turmeric-derived AgNPs might provide their positive effects during development by alleviating oxidative stress.

We observed a direct relationship between pigmentation loss in pupae and adults with the concentration of chemically derived, aloe-vera-derived, and aloe–turmeric-derived AgNPs ingested. However, ingestion of turmeric-derived AgNPs induced pigmentation loss only at a very high dose (i.e., 250 mg/L). These results indicate a superior biological response to the ingestion of turmeric-based AgNPs than that of the ingestion of chemically, aloe- or aloe–turmeric-derived AgNPs.

We found that the ingestion of AgNPs incorporated food throughout aging led to a decline in the climbing ability of flies, which was dependent on the type and concentration of AgNPs and the age of the flies.

At an early age, only moderate doses of chemical AgNPs and aloe vera–turmeric AgNPs lowered the climbing ability. Later, the deterioration in climbing ability became evident with all the types and doses of AgNPs. Therefore, it can be inferred that the negative effects of nanoparticle exposure on the motor deficit could be manifested progressively with aging, and maybe because of in vivo processing of NPs and their accumulation over a period that needs to be assessed in greater detail.

These results suggested a progressively aggravating system-wide toxicity, leading to the death of most flies at high-dose exposures of AG-C and AG-BAT, and hence, highlighted the extensive toxicity associated with these NPs.

However, in the present study, we showed that AgNPs tended to reduce the bio-toxicity when synthesized from scratch with the turmeric extract as the precursor. The effect of curcumin added along with the silver-nanoparticle-rich diet in *Drosophila* was reported earlier [25].

To further understand whether turmeric alone is enough to lead to increased efficacy and lower toxicity in the systems, a separate in vitro test was done by inoculating the HEK-293 culture with only the turmeric extract of the same concentration as the other nanoparticles.

It was observed that using the same concentration of only turmeric extract led to the death of cells. Hence, we concluded that only when turmeric extract is used to synthesize the AgNPs, incorporated with the AgNPs, or is used in dilution, does it lose its toxic effect and helps with cell growth. This, too, is an important finding, as many ethnicities traditionally use turmeric directly for wound healing. The mechanism is yet to be studied.

This study also showed that not all biologically derived AgNPs are safe for biological applications. Their effects depend on specific properties of the phytochemicals that can influence their interaction with silver.

The mechanism of how the plant extracts impart unique properties to nanoparticles is still not fully understood. Furthermore, the mechanism of how nanoparticles interact with cells (in both animals and plants) is also not well understood. Moreover, the NPs that work well for animal/mammalian cells might not do so for plants. Hence an extensive study is needed in this field to have a universally good nanoparticle. We are working on finding these answers in our next project.

## 5. Conclusions

Our studies showed that the use of plant extract during the synthesis process is a good option for a low cost, environmentally friendly, and easy method of preparation of silver nanoparticles. The incorporation of turmeric extract in the synthesis process slowed the growth of NPS, whereas aloe vera enhanced it. The presence of aloe vera extract in the synthesis led to the formation of various oxides of silver. From in vitro studies, we could conclude that the AgNPs synthesized using turmeric extract showed faster and more sustained cell growth as compared with other AgNPs. These NPs also showed no cytotoxicity.

Our studies also showed that though some plant extracts are known for their healing properties (turmeric and aloe vera in our case), the presence of the nanoparticles can either enhance (AG-BT) or undermine (AG-BA) their efficacy. Hence, the use of the right plant extract is essential and there needs to be an extensive study to aid with choosing these extracts.

We also provided strong evidence of a healthy biological response observed upon in vivo exposure to turmeric-derived AgNPs in a dose-dependent manner. Turmeric AgNPs hence may be shown to be a safer alternative to the extensively used chemically synthesized AgNPs. With further detailed toxicity analysis and standardization of synthesis, turmeric-derived AgNPs can be commercialized.

## Figures and Tables

**Table 1:** EDS results showing the wt.% of elemental silver, elemental oxygen, and trace elements (N, Si, etc.) in the synthesized samples.

Sample Name	Silver	Oxygen	Trace Elements
AG-C	74.9	17.6	7.5
AG-BT	17.7	59.4	22.9
AG-BA	90.7	8.5	0.8
AG-BAT	15.5	77	7.5

**Table 2:** DLS and zeta potential measurements of nanoparticles.

Sample	Hydrodynamic Diameter (nm)	Polydispersity Index (PDI)	Zeta Potential (mV)
AG-C (PVP AgNPs)	124.4	0.425	−4.84
AG-BT (turmeric AgNPs)	494.5	0.589	−28.9
AG-BAT (aloe vera–turmeric AgNPs)	778.9	0.724	−23.7
AG-BA (aloe vera AgNPs)	463.2	0.449	−15.35

## Data Availability

Not applicable.
